# Fat Embolism

**Published:** 1917-04

**Authors:** 


**Fat Embolism** is emphasized as the cause of many cases of military shock by W. T. Porter of Boston M. & S. Jour., Feb. 15. Shock has been observed in the present war to be especially frequent after shell fractures of the femur and, more generally, to be associated with wounds by such missiles as tend to shatter bones, in proportion as the amount of medullary tissue involved suggests the possibility of fat embolism. Shock with fall of blood pressure may be produced experimentally by injecting fat into a vein, but the presence of fat, per se, in the blood does not cause such symptoms, embolism being requisite.

